# Cesarean Scar Pregnancy Diagnosed in the First Trimester: A Report of Two Cases and Literature Review

**DOI:** 10.7759/cureus.106730

**Published:** 2026-04-09

**Authors:** Anibri Mouna, Rim Laaboudi, Benabdeslam Rim, Bargach Samir, Elhassouni Fatema

**Affiliations:** 1 Department of Obstetrics and Gynecology, Souissi Maternity Hospital, Rabat, MAR

**Keywords:** case report, cesarean scar pregnancy, ectopic pregnancy, fertility preservation, transvaginal ultrasound, uterine scar

## Abstract

Cesarean scar pregnancy (CSP) is a rare form of ectopic pregnancy characterized by implantation of the gestational sac within a previous cesarean section scar. Its incidence has increased in parallel with rising global cesarean delivery rates. Early diagnosis is critical to prevent life-threatening complications such as uterine rupture and severe hemorrhage.

We report two first-trimester CSP cases. The first involved a 30-year-old woman with three previous cesarean sections presenting with pelvic pain and hemoperitoneum. Emergency laparotomy with excision of the gestational tissue and uterine scar repair was performed. The second case concerned a 37-year-old woman diagnosed early by transvaginal ultrasound and successfully treated with a two-dose systemic methotrexate regimen, achieving complete biochemical and sonographic resolution. Transvaginal ultrasound is the cornerstone of CSP diagnosis. Management options include systemic or local methotrexate, hysteroscopic or laparoscopic resection, uterine artery embolization, or laparotomy in unstable patients. Treatment must be individualized according to hemodynamic status, gestational age, beta-human chorionic gonadotropin (β-hCG) level, myometrial thickness, and future fertility desire.

CSP requires early recognition and tailored management. These cases illustrate the variability in clinical presentation and emphasize the importance of first-trimester ultrasound evaluation in women with previous cesarean deliveries.

## Introduction

Cesarean scar pregnancy (CSP) is a rare form of ectopic pregnancy in which implantation occurs within the fibrotic tissue of a previous cesarean section scar. Its estimated incidence ranges from approximately 1 in 1,800 to 1 in 2,500 pregnancies and accounts for up to 6% of ectopic pregnancies in women with a history of cesarean delivery [1]. The pathophysiological mechanism is believed to involve trophoblastic invasion through a microscopic myometrial defect at the site of the cesarean scar, allowing implantation within the scar tissue [2]. If left untreated, CSP may lead to severe complications, including uterine rupture, massive hemorrhage, hysterectomy, and significant maternal morbidity.

The diagnosis is primarily established using transvaginal ultrasound based on well-defined sonographic criteria, including an empty uterine cavity and cervical canal, a gestational sac embedded in the anterior lower uterine segment at the level of the cesarean scar, and a thin myometrial layer (<3 mm) between the gestational sac and the bladder [3]. The first case of CSP was reported by Godin et al. in 1997, highlighting both the diagnostic challenge and the potential for severe hemorrhagic complications [4].

In this report, we present two cases illustrating different clinical presentations and management strategies for CSP.

## Case presentation

Case 1

**Figure 1:**
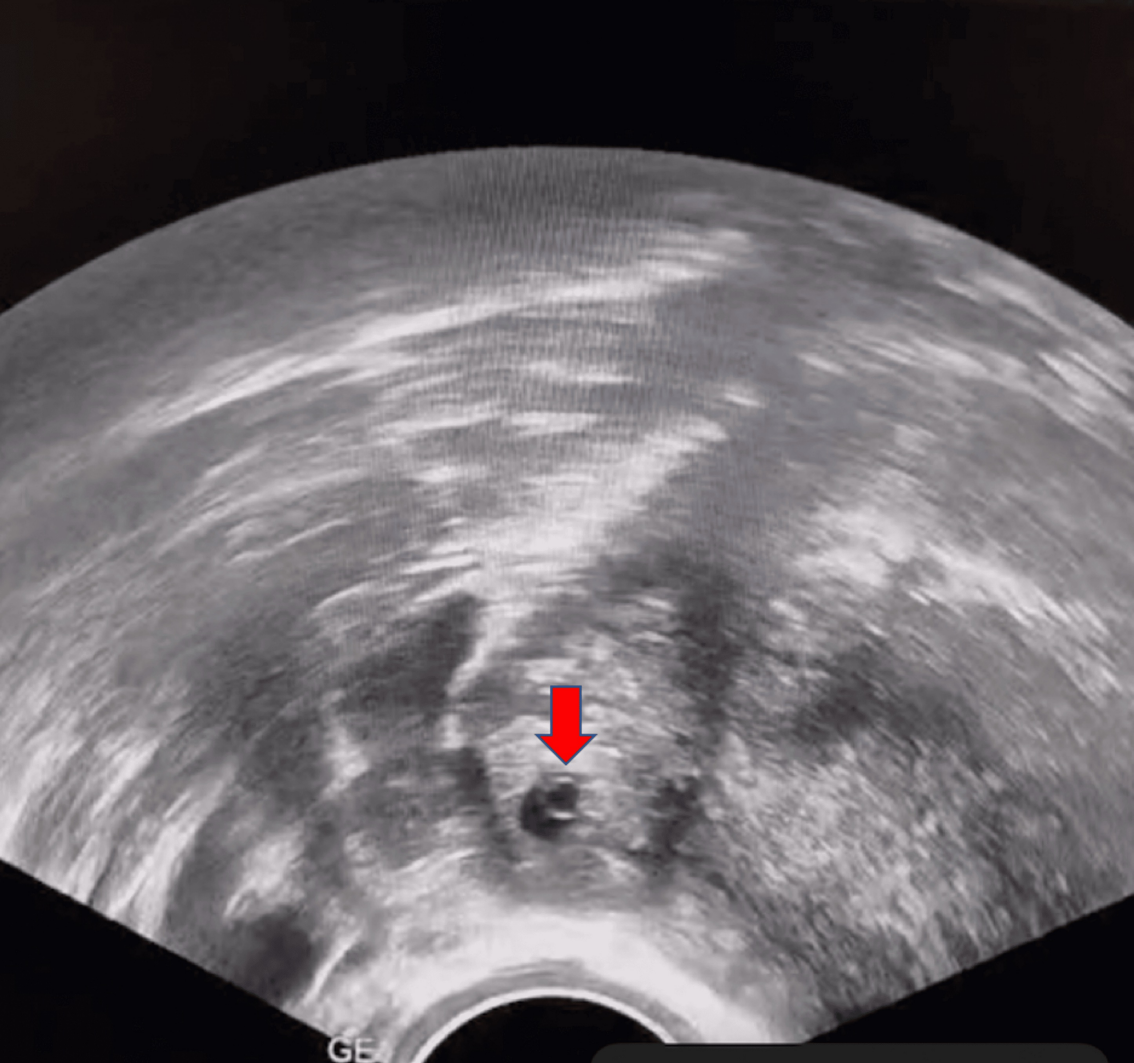
Transvaginal ultrasound revealed a gestational sac embedded in the cesarean scar (red arrow).

**Figure 2:**
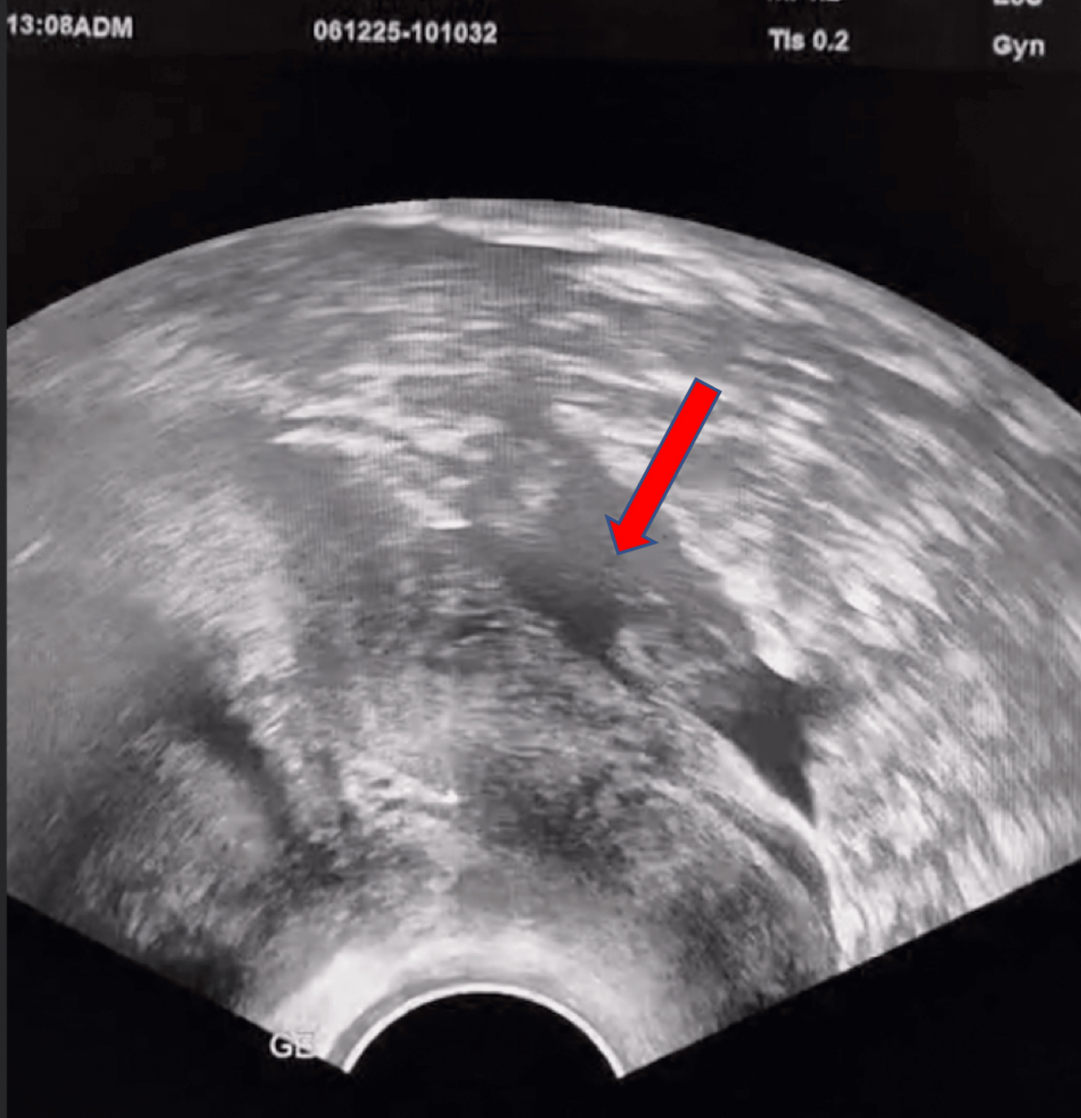
Transvaginal ultrasound revealed a moderate pelvic effusion (red arrow).

A 30-year-old gravida 4, para 3 (G4P3) woman with three prior cesarean sections presented with acute pelvic pain and scant dark metrorrhagia at eight weeks of amenorrhea. She was hemodynamically stable. On examination, uterine enlargement and signs of peritoneal irritation were noted. Transvaginal ultrasound revealed a gestational sac embedded in the cesarean scar (Figure 1), with a yolk sac and moderate pelvic effusion (Figure 2). The residual myometrial thickness between the gestational sac and the bladder measured approximately 2 mm. Given the risk of rupture, emergency laparotomy was performed.

**Figure 3:**
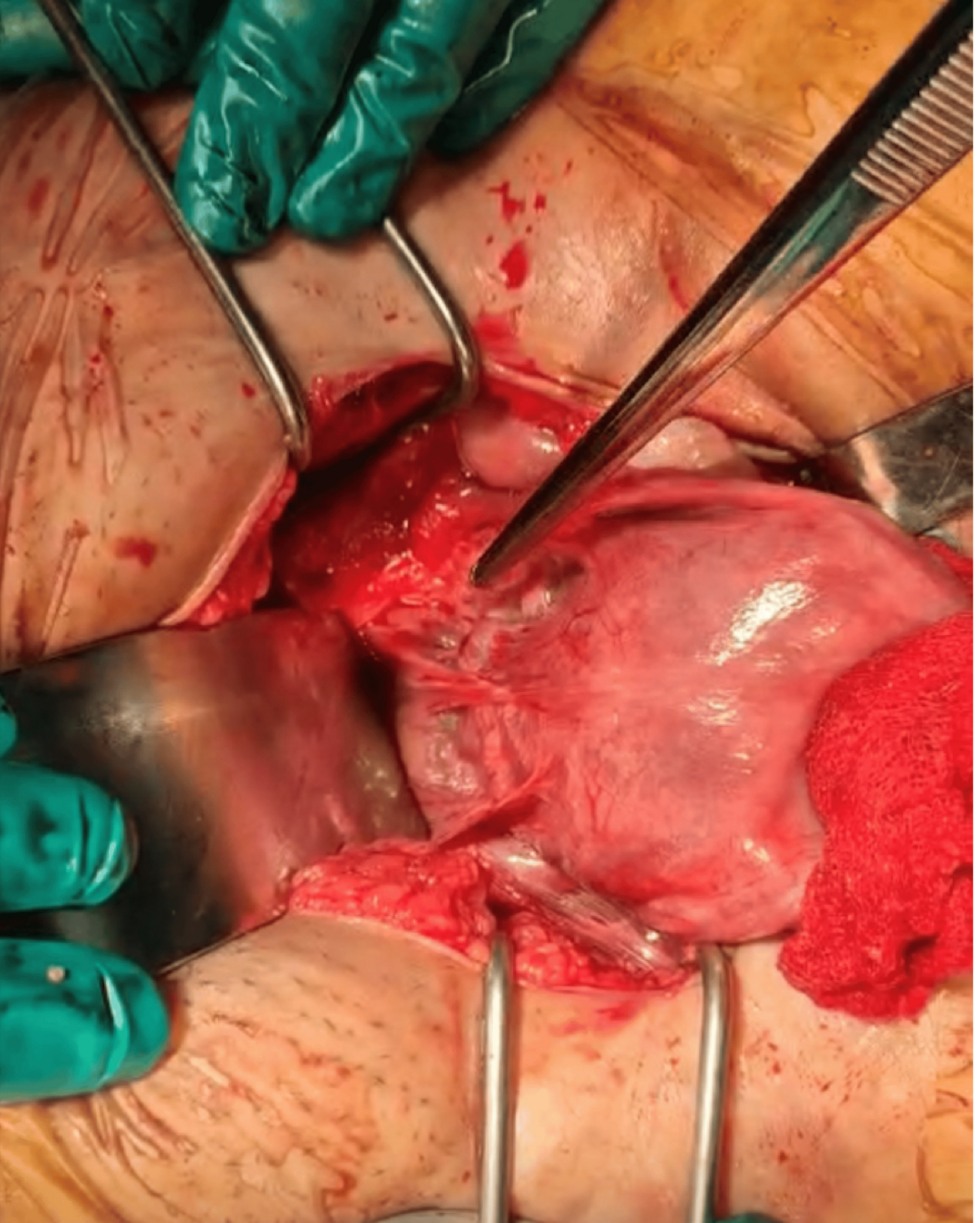
Bluish isthmic mass protruding through the scar area.

**Figure 4:**
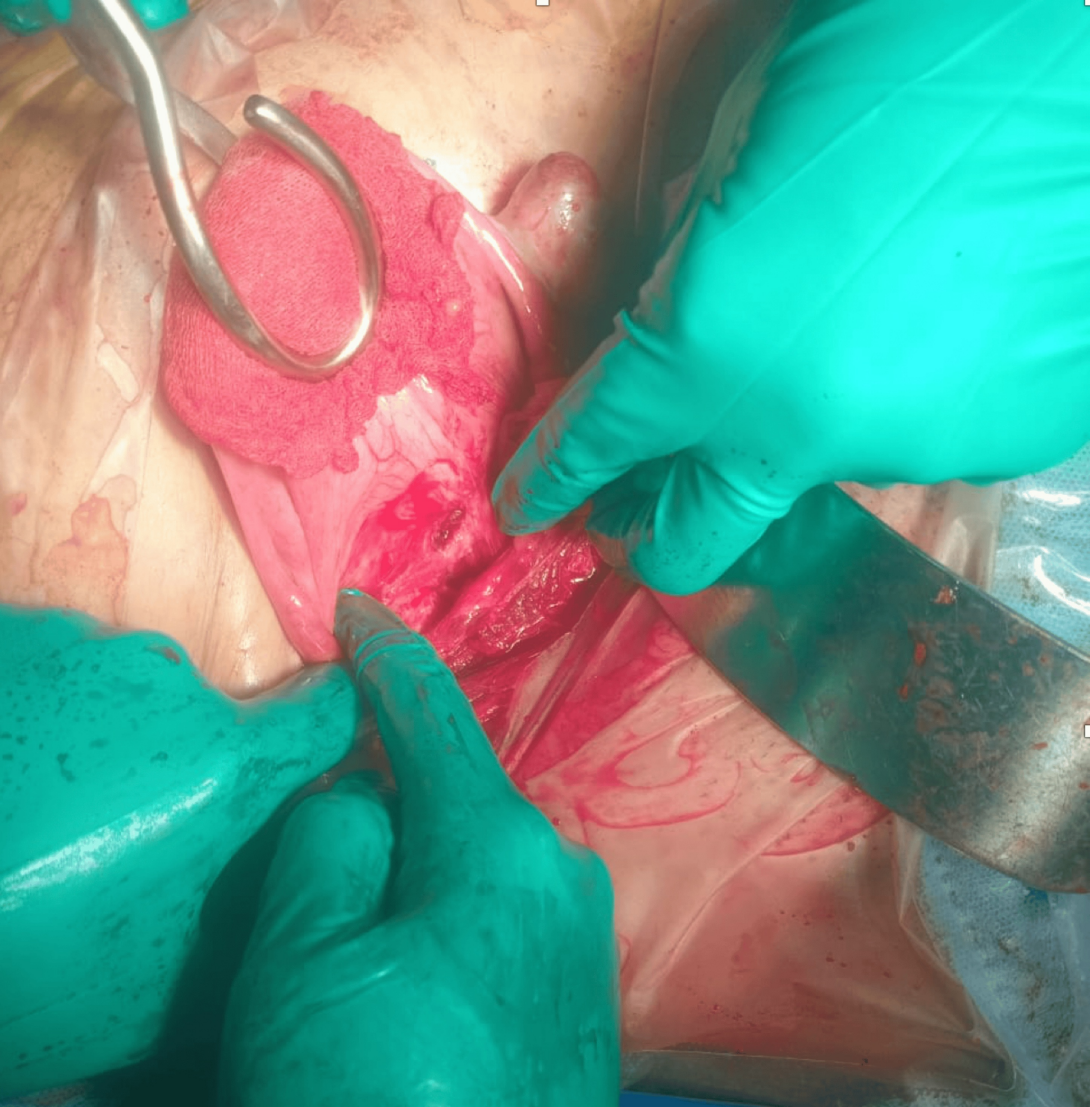
After bladder dissection, the conceptus was removed.

Due to the clinical context, an emergency laparotomy was performed. Exploration revealed a bluish isthmic mass protruding through the scar area (Figure 3). Approximately 300 mL of hemoperitoneum was identified intraoperatively. After bladder dissection, the conceptus was removed, and the cesarean scar defect was repaired by hysterorrhaphy (Figure 4). Postoperative evolution was uncomplicated, and beta-human chorionic gonadotropin (β-hCG) normalized within three weeks.

Case 2

**Figure 5:**
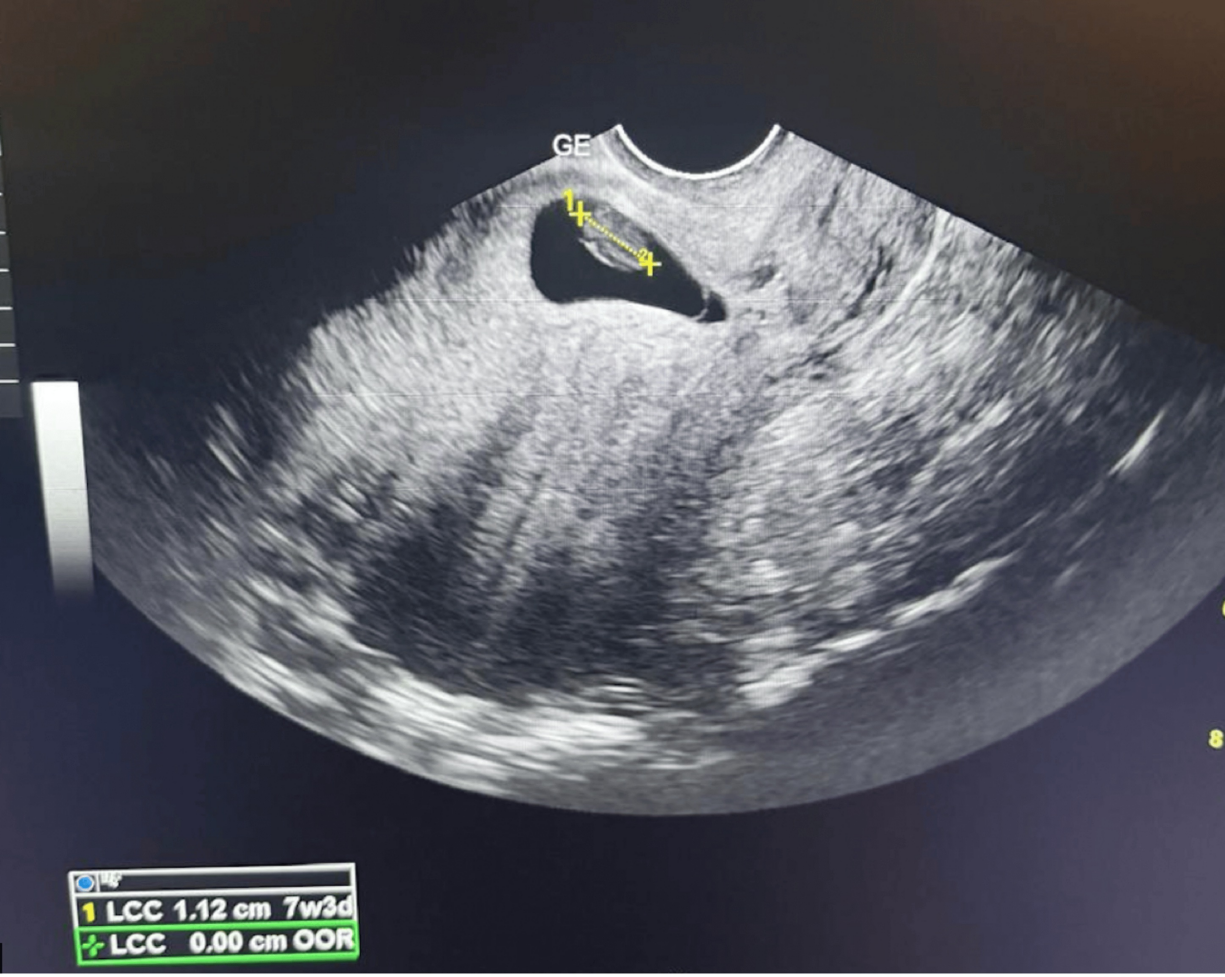
Transvaginal ultrasound showed a gestational sac implanted in the cesarean scar, containing an 11-mm embryo without cardiac activity.

A 37-year-old gravida 2, para 1 (G2P1) woman with one prior cesarean delivery presented with pelvic pain and dark vaginal bleeding at seven weeks of amenorrhea. Transvaginal ultrasound showed a gestational sac implanted in the cesarean scar, containing an 11-mm embryo without cardiac activity (Figure 5). The myometrial thickness was measured at 3 mm, with no evidence of bladder invasion. There was no pelvic effusion.

She received systemic methotrexate. Due to a transient rise in β-hCG, a second dose was administered one week later. Progressive biochemical decline followed, with complete normalization by day 74. Follow-up ultrasound confirmed regression of the avascular mass without bladder involvement.

## Discussion

CSP is classified into two types according to the direction of gestational sac growth. Type I (endogenic) grows toward the uterine cavity and may occasionally progress toward a viable pregnancy, whereas Type II (exogenic) grows toward the serosal surface and is associated with a higher risk of uterine rupture and massive hemorrhage [3,5]. Early diagnosis is essential to prevent severe maternal complications. Transvaginal ultrasound remains the first-line diagnostic modality and demonstrates high diagnostic accuracy when performed by experienced operators [2,3].

Management of CSP is challenging due to the absence of universally accepted guidelines. Treatment options include medical therapy, surgical intervention, or a combination of both, and should be individualized based on gestational age, serum β-hCG levels, the presence of fetal cardiac activity, hemodynamic stability, and the patient’s desire for future fertility [6-9]. Medical management with systemic or local methotrexate is commonly reported in early gestations among hemodynamically stable patients, although success rates vary depending on initial β-hCG and embryonic cardiac activity [9].

Surgical management is indicated in cases of failed medical therapy, advanced gestation, or hemodynamic instability. Options include hysteroscopic resection, laparoscopic excision with scar repair, or laparotomy in emergency situations [10,11].

Systematic first-trimester ultrasound screening in women with previous cesarean delivery is strongly recommended to allow timely, fertility-preserving intervention [6-8]. Although fertility is generally preserved after successful treatment, subsequent pregnancies remain at increased risk for recurrent CSP and placenta accreta spectrum disorders [12,13]. In selected cases, magnetic resonance imaging may be used to evaluate myometrial thickness, bladder invasion, or complex anatomical relationships [14]. Ash et al. reviewed the challenges and options for CSP management, highlighting both medical and surgical strategies [15]. Scar repair during surgery may reduce the risk of recurrence and abnormal placentation in future pregnancies [16]. Subsequent pregnancies remain at increased risk for abnormal placentation, as CSP and early placenta accreta share common histology [17]. Historically, CSP was first described in the late 1990s, and increased awareness has improved early diagnosis over time [4,18].

## Conclusions

CSP is a rare but potentially life-threatening ectopic pregnancy. Early diagnosis through first-trimester transvaginal ultrasound is crucial to prevent severe complications such as uterine rupture and massive hemorrhage. Management should be individualized, taking into account gestational age, clinical presentation, hemodynamic stability, and future fertility desires. Both medical and surgical approaches can be effective when applied to appropriately selected patients. The present cases highlight the importance of early screening and tailored management strategies, emphasizing that timely diagnosis allows fertility-preserving treatment and reduces the risk of catastrophic maternal outcomes.
